# A New Intraoperative Method for Detecting Fistulas in Patients With Peritoneal Dialysis-Associated Pleuroperitoneal Communication

**DOI:** 10.1016/j.atssr.2025.04.011

**Published:** 2025-05-12

**Authors:** Yu Suyama, Tomonari Kinoshita, Ai Otani, Yo Tsukamoto, Takamasa Shibazaki, Takeo Nakada, Takashi Ohtsuka

**Affiliations:** 1 Division of Thoracic Surgery, Department of Surgery, The Jikei University School of Medicine, Tokyo, Japan

## Abstract

Patients undergoing peritoneal dialysis may experience pleuroperitoneal communication caused by increased intraabdominal pressure from dialysis fluid. Even if the fistula is surgically closed, recurrence is common, sometimes requiring a switch to hemodialysis. One cause of recurrence is the difficulty in identifying the microfistula intraoperatively. During thoracoscopic fistula closure in the patient described in this case report, indigo carmine was used to identify the fistula and indocyanine green was then used to confirm closure. As a result of using this efficient and reliable method, pleuroperitoneal communication has not recurred for 2 months postoperatively.

Pleuroperitoneal communication is a rare complication of continuous ambulatory peritoneal dialysis (CAPD).^1^ Although surgical intervention is generally preferred, identifying the fistula intraoperatively can be challenging. Various methods have been proposed, including checking for air leakage,^2^ laparoscopy,^3^ and staining with indigo carmine or indocyanine green (ICG).^4^ However, these methods are imperfect because the fistula may remain undetected or incompletely closed. As a result, patients must switch to hemodialysis, thus increasing their hospital visits and overall burden. We have developed a novel, reliable, and efficient thoracoscopic fistula closure strategy using indigo carmine and ICG.

A 72-year-old woman presented to our hospital (The Jikei University Hospital, Tokyo, Japan) with shortness of breath and a persistent cough lasting several days. Her medical history included chronic renal failure secondary to immunoglobulin A nephropathy, and she had been undergoing CAPD for 3 months. A chest roentgenogram revealed a large right pleural effusion (Figure 1A). Peritoneal scintigraphy confirmed right-sided pleuroperitoneal communication (Figure 1B), and she was scheduled for thoracoscopic surgery to close the fistula.

**Figure 1:**
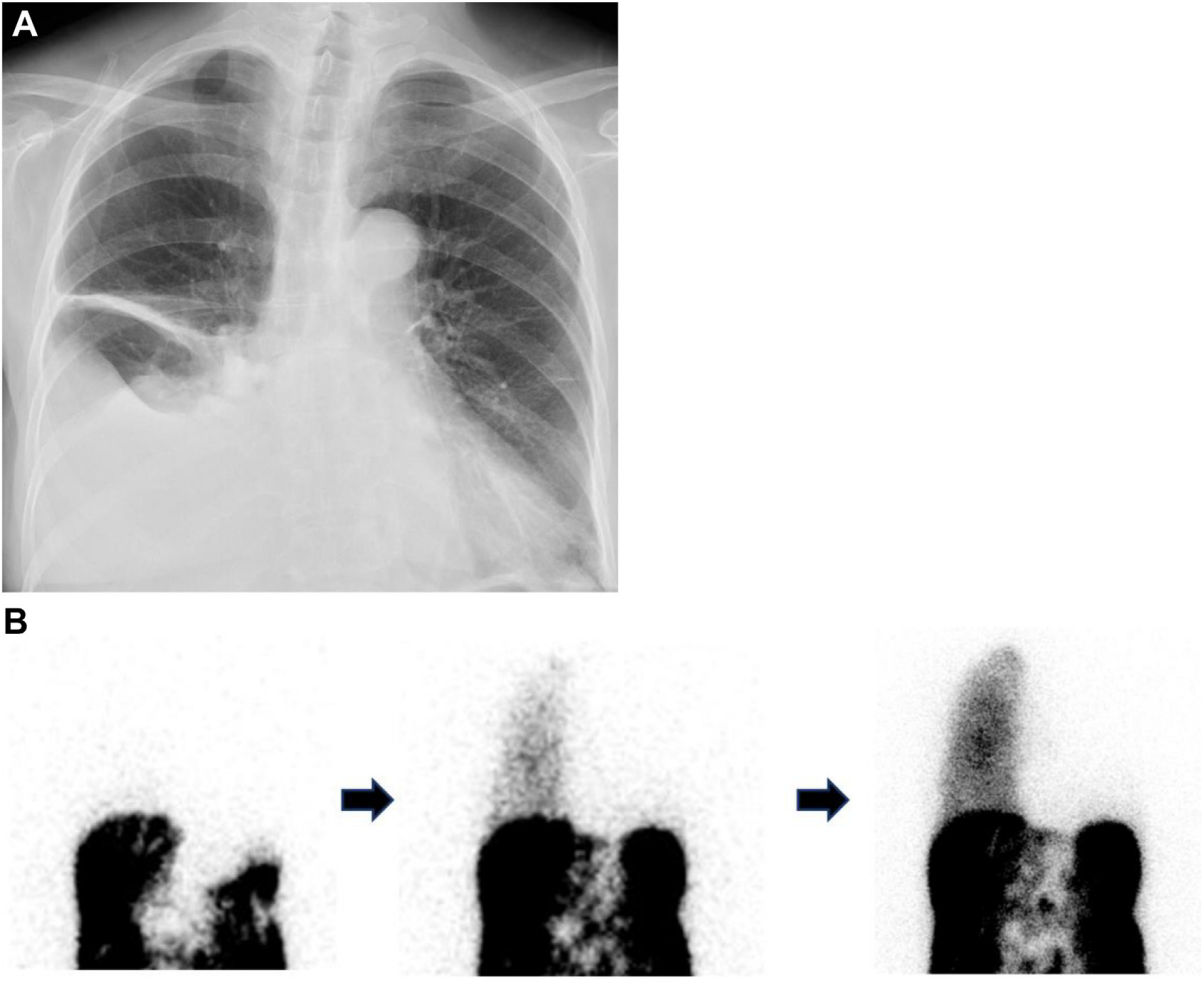
(A) Chest roentgenogram on admission shows right pleural effusion. (B) Peritoneal scintigraphy shows an infusion into the right thoracic cavity.

Video-assisted thoracoscopic surgery was performed using a Stryker 1688 AIM 4K camera (Stryker Corp). Pleural effusion was present at the start of the procedure, but no apparent fistula was identified. Through a Tenckhoff catheter, 1000 mL of peritoneal dialysis (PD) fluid mixed with 20 mg of indigo carmine (a dark blue dye) was infused. Immediately, dark blue-stained pleural effusion was observed (Figure 2A). A fistula was identified on the diaphragm, with no additional fistulas detected (Figure 2B). We resected a portion of the diaphragm, including the fistula site, by using a stapler with reinforcement material (Figure 2C). To confirm closure and check for other fistulas, 2000 mL of dialysate mixed with 25 mg of ICG was infused. No fluorescent dialysate leakage was observed from the suture site or diaphragm in the SPY overlay mode (Figure 2D). Pathologic examination ruled out malignancy or other diseases as the cause of the fistula. CAPD was resumed on postoperative day 7, and no recurrence was observed for 3 months.

**Figure 2:**
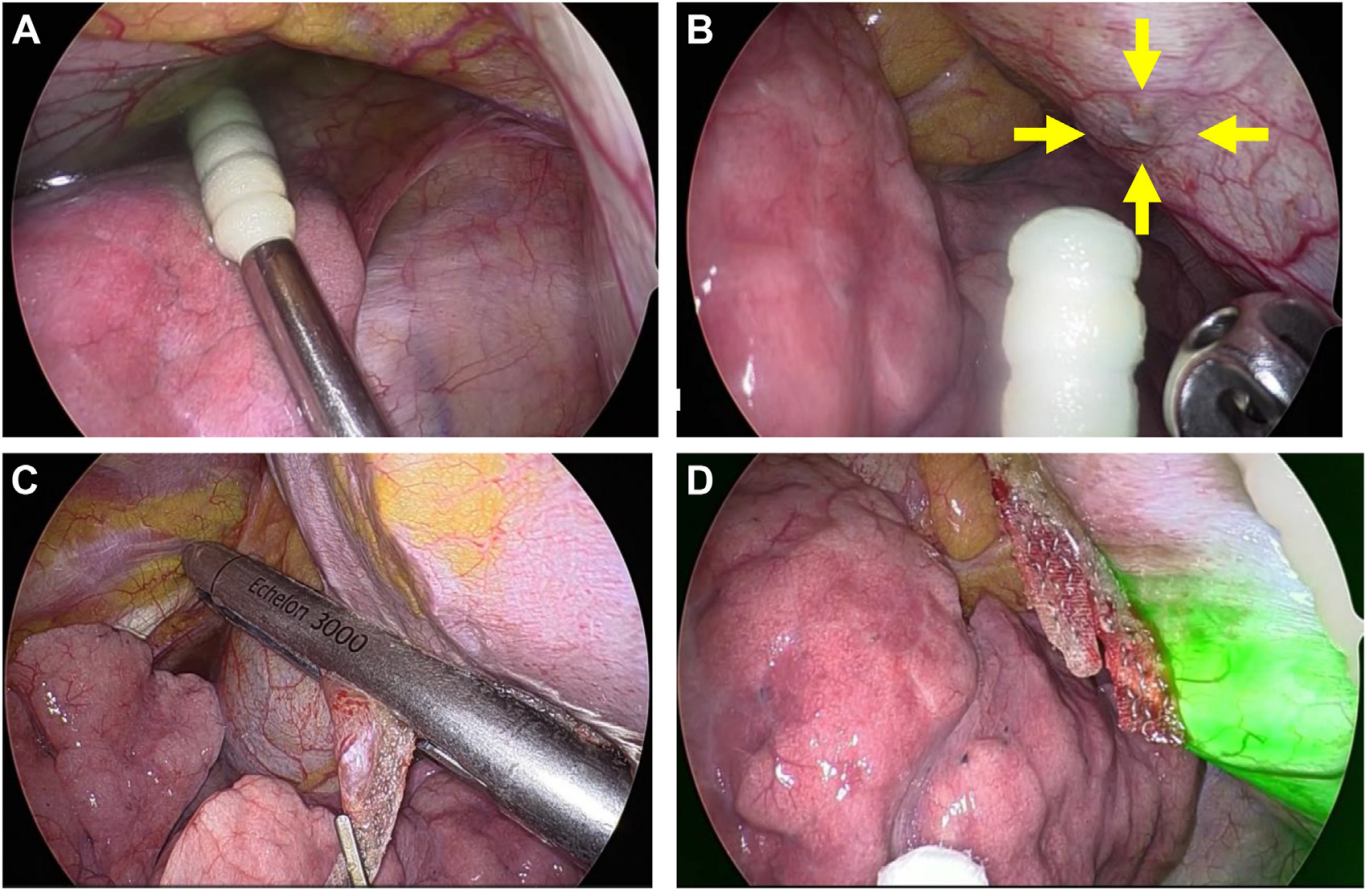
(A) Pleural effusion stained blue with indigo carmine. (B) A diaphragmatic fistula was observed (arrows). (C) The fistula was closed using an automatic suture device with reinforcement material. (D) No leakage of indocyanine green–mixed dialysate into the thoracic cavity was found.

## Comment

Reports indicate that 1.6% of patients undergoing PD experience a pleuroperitoneal fistula.^1^ Although surgical closure is typically performed to allow continued PD, identifying the fistula macroscopically intraoperatively is rare, and postoperative recurrence remains a concern. The success rate of thoracoscopic closure for pleuroperitoneal fistulas ranges from 67% to 90%, depending on whether the fistula is identified intraoperatively.^5^ One study reported an 81% success rate when the fistula was found but only 38% when it was not.

Several methods exist for fistula detection, including carbon dioxide insufflation into the abdominal cavity and infusion of dialysis fluid mixed with indigo carmine or ICG.^4^^,^^6^ However, few studies have introduced efficient techniques for both identifying and confirming fistula closure. We propose a 2-step approach: first, injecting indigo carmine to identify the fistula, followed by using ICG to confirm closure and check for additional fistulas. This report describes the closure of diaphragmatic fistulas by using this dye combination.

In this case, because the fistula was identified using the first 1000 mL of dialysis fluid mixed with indigo carmine, no additional dialysis fluid was added. During regular PD, this patient uses 1500 mL of dialysis fluid. If the fistula could not be identified with the first 1000 mL of dialysate mixed with indigo carmine, the same dialysate was supposed to be added. Conversely, we confirmed that there was no fistula by using 2000 mL of the dialysis fluid mixed with 25 mg of ICG. During both examinations, we maintained the patient in the Trendelenburg position long enough to apply sufficient pressure to the abdomen and diaphragm.

This method leverages fluorescent dyes and endoscopy, which have been widely used in recent surgery. Indigo carmine provides clear visual identification of the fistula site, whereas ICG confirms closure. If ICG is injected first, followed by indigo carmine, the overlapping colors may obscure fistula closure assessment. As a pitfall, if another fistula is identified and closed on the basis of the ICG test for fistula closure, it may be difficult to distinguish between the ICG that previously leaked into the thoracic cavity and the ICG that was administered for reidentification. However, this technique is applicable to multiple fistulas, is cost effective, is minimally invasive, and ensures safe and reliable fistula closure. This technique can be applied not only to pleuroperitoneal communication but also to diseases that require the identification and treatment of multiple fistulas.

## References

[1] Nomoto Y., Suga T., Nakajima K. (1989). Acute hydrothorax in continuous ambulatory peritoneal dialysis--a collaborative study of 161 centers. Am J Nephrol.

[2] Matsuoka N., Yamaguchi M., Asai A. (2020). The effectiveness and safety of computed tomographic peritoneography and video-assisted thoracic surgery for hydrothorax in peritoneal dialysis patients: a retrospective cohort study in Japan. PLoS One.

[3] Yen H.T., Lu H.Y., Liu H.P., Hsieh M.J. (2005). Video-assisted thoracoscopic surgery for hydrothorax in peritoneal dialysis patients - check-air-leakage method. Eur J Cardiothorac Surg.

[4] Hashimoto T., Osaki T., Oka S., Fujikawa T. (2023). Thoracoscopic and laparoscopic approach for pleuroperitoneal communication under peritoneal dialysis: a report of four cases. Surg Case Rep.

[5] Christine Argento A., Kim A., Knauert-Brown M. (2014). Recurrent hydrothorax and surgical diaphragmatic repair: report of 2 cases and review of the literature. J Bronchology Interv Pulmonol.

[6] Manabe T., Ono K., Oka S., Kawamura Y., Osaki T. (2021). A case of pleuroperitoneal communication in which establishing a laparoscopic pneumoperitoneum was useful for the detection of a fistula. Surg Case Rep.

